# Lung microvascular occlusion by platelet-rich neutrophil-platelet aggregates promotes cigarette smoke–induced severe flu

**DOI:** 10.1172/jci.insight.167299

**Published:** 2024-01-23

**Authors:** Tomasz W. Kaminski, Tomasz Brzoska, Xiuying Li, Ravi Vats, Omika Katoch, Rikesh K. Dubey, Kamal Bagale, Simon C. Watkins, Bryan J. McVerry, Tirthadipa Pradhan-Sundd, Lianghui Zhang, Keven M. Robinson, Toru Nyunoya, Prithu Sundd

**Affiliations:** 1 Thrombosis and Hemostasis Program, VERSITI Blood Research Institute, Milwaukee, Wisconsin, USA.; 2 Pittsburgh Heart, Lung and Blood Vascular Medicine Institute (VMI),; 3 Division of Hematology and Oncology, and; 4 Division of Pulmonary Allergy and Critical Care Medicine, University of Pittsburgh School of Medicine, Pittsburgh, Pennsylvania, USA.; 5 Department of Bioengineering,; 6 Center for Biologic Imaging, and; 7 Department of Cell Biology, University of Pittsburgh, Pittsburgh, Pennsylvania, USA.; 8 Transfusion Medicine, Vascular Biology and Cell Therapy Program, VERSITI Blood Research Institute, Milwaukee, Wisconsin, USA.; 9 Center for Vaccine Research, University of Pittsburgh, Pittsburgh, Pennsylvania, USA.

**Keywords:** Infectious disease, Pulmonology, Influenza, Neutrophils, Platelets

## Abstract

Cigarette smoking is associated with a higher risk of ICU admissions among patients with flu. However, the etiological mechanism by which cigarette smoke (CS) exacerbates flu remains poorly understood. Here, we show that a mild dose of influenza A virus promotes a severe lung injury in mice preexposed to CS but not room air for 4 weeks. Real-time intravital (in vivo) lung imaging revealed that the development of acute severe respiratory dysfunction in CS- and flu-exposed mice was associated with the accumulation of platelet-rich neutrophil-platelet aggregates (NPAs) in the lung microcirculation within 2 days following flu infection. These platelet-rich NPAs formed in situ and grew larger over time to occlude the lung microvasculature, leading to the development of pulmonary ischemia followed by the infiltration of NPAs and vascular leakage into the alveolar air space. These findings suggest, for the first time to our knowledge, that an acute onset of platelet-driven thrombo-inflammatory response in the lung contributes to the development of CS-induced severe flu.

## Abstract 2

<p>Platelet-rich aggregates promote cigarette smoke-induced severe flu.</p>

## Graphical abstract

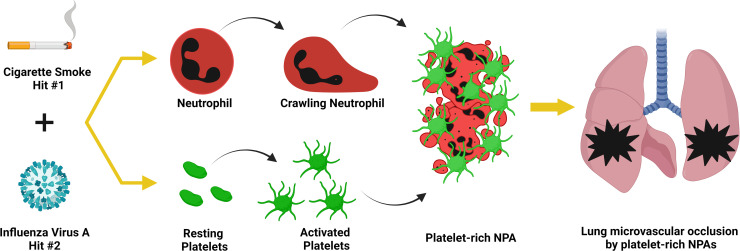

## Introduction

Flu is an infectious respiratory disease caused by the Influenza A viruses (IAV) that affects ~35 million people every year in the United States (1–4). The majority of patients with the flu present with fever, myalgia, fatigue, and upper respiratory tract symptoms, and the IAV infection is usually self-resolving (5). However, a more severe disease can occur involving inflammation of the lower respiratory tract, leading to the development of pneumonia, respiratory failure, and even death (5, 6). Unfortunately, both antiviral therapy and flu vaccines benefit only a limited number of patients with the flu (1, 2, 7), and the severe flu continues to result in over ~380,000 hospitalizations and ~28,000 deaths each year in the United States (1–4).

Cigarette smoking, one of the most prevalent addictions affecting over ~30 million adults in the United States, is a major risk factor for development of severe flu (8–12). Cigarette smoke (CS) contains numerous chemical compounds that significantly increase the risk of lung diseases by promoting immune dysregulation (13, 14). Although recent evidence supports a role for the thrombo-inflammation in flu (15–19), the etiological mechanism underlying cigarette smoking–induced flu severity remains poorly understood (13, 20–22). Identifying the cellular and molecular mechanism driving CS exposure–induced severe flu will enable the development of new therapies to prevent acute lung injury (ALI) in smokers with the flu.

Here, we used a unique 2-hit model of CS exposure followed by a mild-dose of intranasal IAV infection in mice (20, 21, 23, 24) that led to more severe lung injury than IAV infection alone. Using quantitative fluorescence intravital lung microscopy (qFILM) in live mice (25–29), we reveal for the first time to our knowledge that the prior exposure to CS exacerbates flu-induced lung injury by promoting lung microvascular occlusion by large platelet-rich neutrophil-platelet aggregates (NPAs), leading to the development of pulmonary ischemia followed by vascular leakage. The current study is the first to our knowledge to identify a role for platelet-driven thrombo-inflammatory response in CS-induced flu severity.

## Results

### Four-week CS exposure followed by flu promotes severe lung injury in mice.

**Figure 1:**
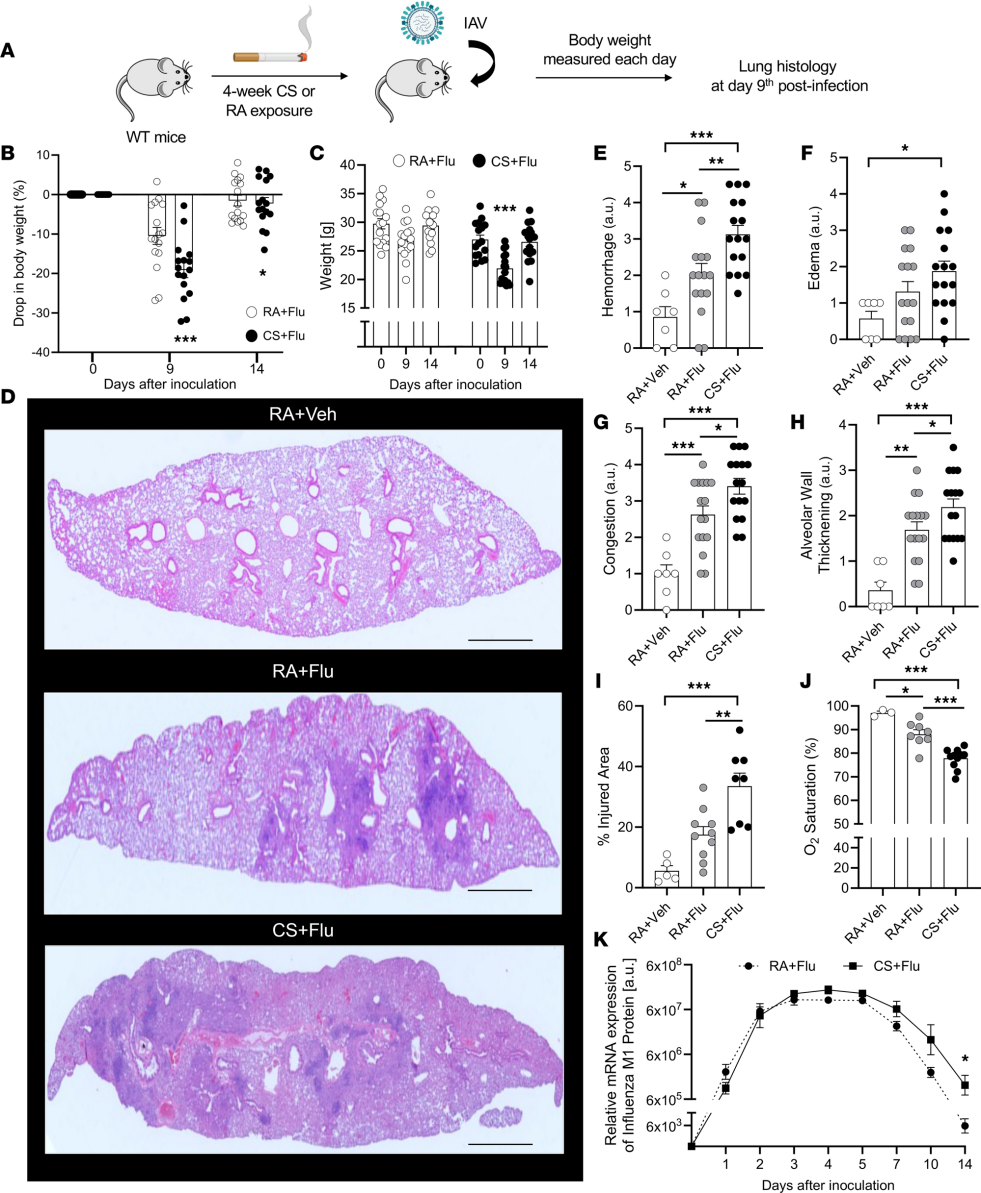
CS+Flu promotes severe lung injury in mice. (**A**) Experimental scheme. WT mice exposed to cigarette smoke (CS) or room air (RA) for 4 weeks followed by intranasal inoculation with influenza A virus (flu) or sterile PBS as vehicle (Veh). Body weight was measured every day after inoculation for 14 days, and lung injury was assessed at day 9 after inoculation. (**B** and **C**) Percent drop in body weight and absolute body weight at day 0, 9, and 14 after inoculation in RA+Flu and CS+Flu mice (*n* = 16 per group). (**D**) Representative H&E-stained histological sections of the whole left lung of an RA+Veh, RA+Flu, and CS+Flu mouse at day 9 after inoculation. Scale bars: 100 μm. (**E**–**J**) Lung histological sections were scored (refer to Methods for details, *n* = 8–16 per group) for severity of hemorrhage (**E**), pulmonary edema (**F**), vascular congestion (**G**), alveolar wall thickening (**H**), percentage of injured area (**I**), and percent blood oxygen saturation in RA+Veh, RA+Flu and CS+Flu mice (**J**) (*n* = 3–12 per group) at day 4 after inoculation. (**K**) Relative mRNA expression of Influenza M1 Protein (refer Methods for details) at different days after flu infection in RA+Flu and CS+Flu mice (*n* = 3–5 per group). Data are shown as mean ± SEM and compared using Student’s *t* test. Comparative statistical analysis was performed by 1-way ANOVA. Comparative statistical analysis was performed by 1-way ANOVA with Bonferroni correction. *P < 0.05; **P < 0.01; ***P < 0.001.

WT mice on a C57BL/6J background were exposed to CS or room air (RA) for 4 weeks, followed by intranasal administration of sterile PBS (vehicle) or a mild dose of IAV (experimental scheme shown in Figure 1A). Based on the prior studies, an approximately 15% or higher drop in body weight within 8–9 days of IAV infection is a benchmark of severe flu in mice (19, 24, 30, 31). In our current 2-hit mouse model, the IAV dose was titrated to achieve a ~10% drop in body weight in the RA+Flu (flu only) group at day 9 after IAV infection (Figure 1B); however, the same IAV dose led to a ~20% drop in body weight in the CS+Flu group at day 9 after IAV infection (Figure 1B), suggestive of the development of severe flu in CS+Flu compared with the RA+Flu group. Indeed, the body weight at day 9 after IAV infection was significantly reduced only in the CS+Flu but not RA+Flu group (Figure 1C). However, identical to the prior reports (19, 24, 30, 31), the body weight recovered back to the preinfection levels following resolution of infection in both the groups by day 14 (Figure 1C). Next, H&E-stained histological sections of the whole left lung were analyzed at day 9 after IAV infection to compare the severity of ALI using the guidelines set by the American Thoracic Society (32, 33). As shown by the representative histological images (Figure 1D) and the corresponding scoring analyses (Figure 1, E–I), the development of severe ALI in the CS+Flu group was confirmed by the significantly higher alveolar hemorrhage (Figure 1E), pulmonary edema (Figure 1F), pulmonary vascular congestion (Figure 1G), alveolar wall thickening (Figure 1H), and percent injured area (Figure 1I) in the CS+Flu compared with the RA+Flu group. The development of severe ALI in the CS+Flu group was further supported by the significantly lower blood oxygen saturation (SpO_2_; severe hypoxemia) in the CS+Flu (SpO_2_ = 77.8% ± 1.3%) compared with the RA+Flu (SpO_2_ = 88.1% ± 1.9%) group within 4 days of IAV infection (Figure 1J), which was not associated with difference in lung viral burden (mRNA levels of IAV M1 protein shown in Figure 1K or plaque-forming units [pfu] per gram of lung tissue shown in Supplemental Figure 2; supplemental material available online with this article; https://doi.org/10.1172/jci.insight.167299DS1). The viral burden was different between the 2 groups only at day 14 after IAV infection (Figure 1K); however, this time point corresponds to complete recovery of body weight (Figure 1, B and C) and low levels of viral load (Figure 1K) in both RA+Flu and CS+Flu groups. Histological analysis of RA+Vehicle (Veh; without CS or flu) group showing absence of lung injury is included for reference in Figure 1, D–J. Importantly, mice exposed to 4 weeks of CS only (without IAV infection) did not develop any lung injury (Supplemental Figure 1).

### Early onset of thrombo-inflammation promotes severe lung injury in CS+Flu mice.

**Figure 2:**
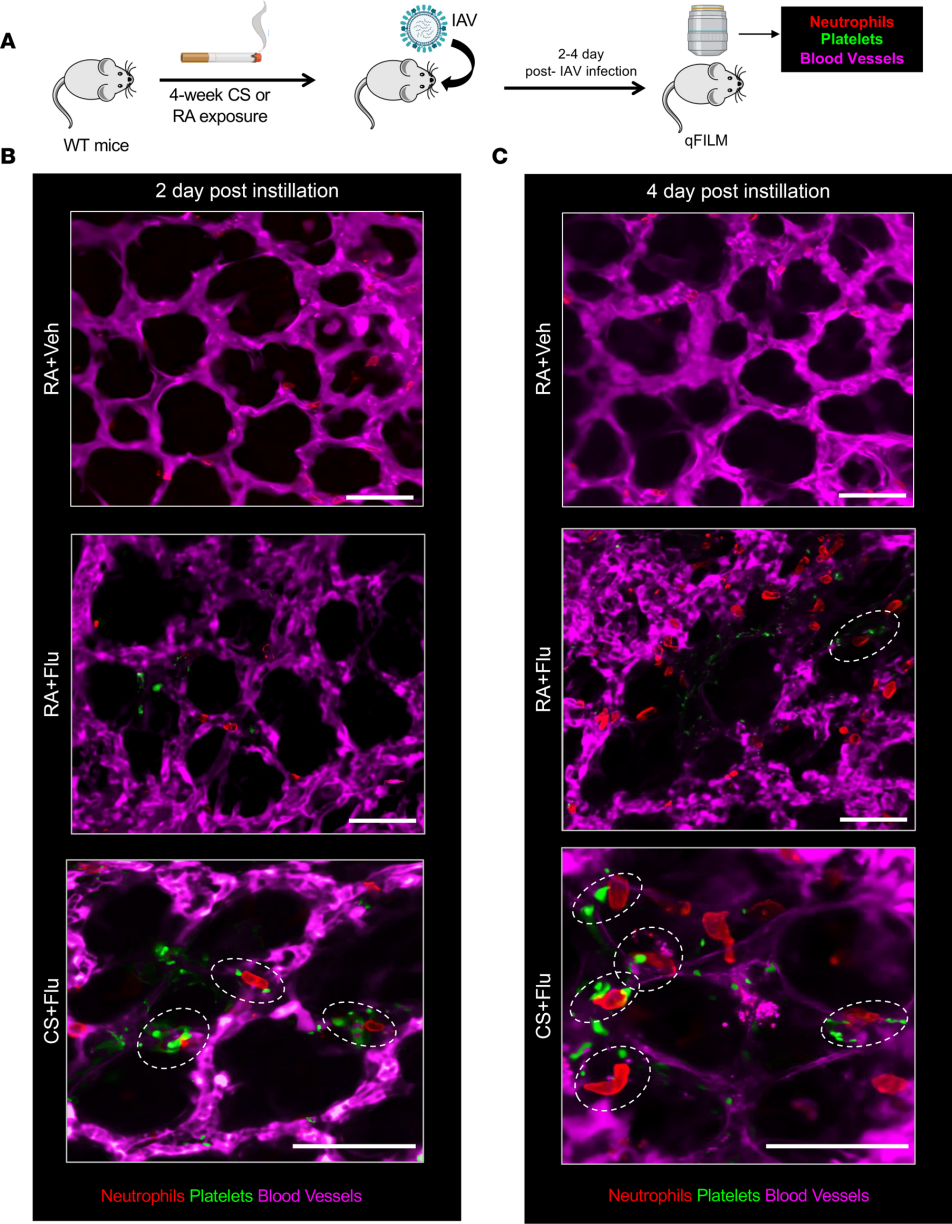
CS+Flu promotes early-onset of thrombo-inflammation in mice. (**A**) Experimental scheme. WT mice exposed to cigarette smoke (CS) or room air (RA) for 4 weeks followed by intranasal inoculation with influenza A virus (flu) or sterile PBS as vehicle (Veh), and quantitative fluorescence intravital lung microscopy (qFILM) was used to assess thrombo-inflammation in the lung of live mice at 2 and 4 days after inoculation. The microcirculation (pseudo-colored purple), neutrophils (red), and platelets (pseudo-colored green) were labeled in vivo by i.v. administration of FITC dextran, AF546 anti–mouse Ly6G Ab, and V450 anti–mouse CD49b Ab, respectively. Refer to Methods for details. (**B** and **C**) Representative qFILM images of lung microcirculation in RA+Veh, RA+Flu, and CS+Flu mice are shown at (**B**) day 2 and (**C**) day 4 after flu infection. Neutrophil-platelet aggregates (NPAs) are marked by dashed ovals. Erythrocytes are visible as dark cells within the lung microcirculation. Complete time series for images in **B** and **C** are shown in Supplemental Videos 1–6. Scale bars: 50 μm.

Next, qFILM was conducted in live mice to assess the role of thrombo-inflammation in development of severe hypoxemia in CS+Flu compared with RA+Flu mice within 4 days of IAV infection. Experimental scheme is shown in Figure 2A. Erythrocytes (dark cells) and neutrophils (red) were rapidly transiting through the pulmonary microcirculation (purple), suggestive of unobstructed blood flow in the lung of mice exposed to RA+Veh at both day 2 (Figure 2B [top panel] and Supplemental Video 1) and day 4 (Figure 2C [top panel] and Supplemental Video 2) after intranasal administration of vehicle. In mice exposed to RA+Flu, erythrocytes (dark cells) were rapidly transiting, and neutrophil (red) recruitment was absent in the pulmonary microcirculation (purple) at day 2 after IAV-infection (Figure 2B [middle panel] and Supplemental Video 3). However, neutrophil (red) accumulation and formation of NPAs (red and green colocalization) were evident in RA+Flu mice at day 4 after IAV infection (an NPA marked with dotted ellipse in Figure 2C [middle panel] and Supplemental Video 4). Unlike in the mice exposed to RA+Flu, NPAs (marked with dotted ellipses) were found to be sequestered within the pulmonary microvessels even at day 2 after IAV infection (Figure 2B [lower panel] and Supplemental Video 5) and grew in numbers by day 4 after IAV infection (Figure 2C [lower panel] and Supplemental Video 6) in the CS+Flu mice. The quantitative analysis of qFILM data revealed that the number of NPAs per field of view (FOV; size = 65,000 μm^2^) were significantly higher in CS+Flu compared with RA+Flu mice at both day 2 and 4 after infection (Supplemental Figure 3).

### Platelet-rich NPAs form in situ to enable pulmonary thrombo-inflammation in CS+Flu mice.

**Figure 3:**
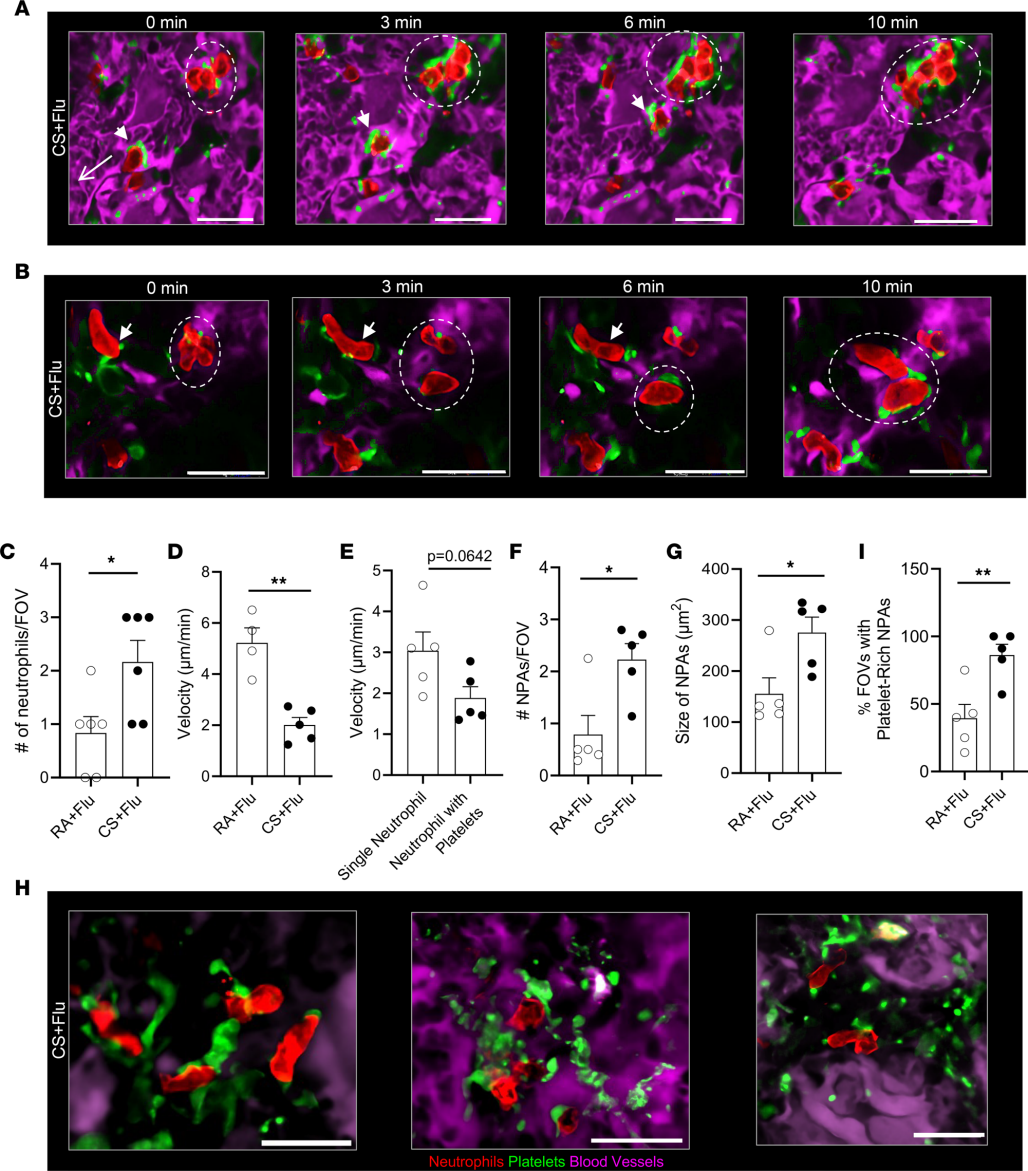
In situ formation of large platelet-rich NPAs promotes pulmonary thrombo-inflammation in CS+Flu mice. Mice were exposed to CS or RA for 4 weeks followed by inoculation with flu, and qFILM was used to assess thrombo-inflammation in the lung at 4 days after infection. Microcirculation (pseudocolored purple), neutrophils (red), and platelets (pseudocolored green). (**A** and **B**) qFILM images of 2 separate field of views (FOVs) in the lung of CS+Flu mice at four different time points. (**A**) A neutrophil bound to platelets (arrowhead) crawls intravascularly to join a large NPA (dashed ellipse). Refer to Supplemental Video 7. Arrow denotes direction of blood flow. Scale bar: 25 µm. (**B**) A neutrophil bound to platelets (arrowhead) crawls intravascularly to join an existing NPA (dashed ellipse). Scale bar: 20 µm. Refer to Supplemental Video 8. qFILM analysis revealed that (**C**) the number of neutrophils crawling toward a large NPA per FOV was significantly higher and (**D**) the crawling velocity of neutrophils was significantly lower in the lung microcirculation of CS+Flu than RA+Flu mice. (**E**) The crawling velocity of single neutrophils (without bound platelets) was not different from the neutrophils bound to platelets. qFILM images were analyzed to compare (**F**) number of NPAs per FOV and (**G**) size of NPAs in the lung of RA+Flu and CS+Flu mice. (**H**) Three representative qFILM images of platelet-rich NPAs in the lung microcirculation of CS+Flu mice. Scale bar: 10 μm (left) and 25 μm (middle and right). (**I**) Percent FOVs containing at least 1 platelet-rich NPA in the lung of RA+Flu and CS+Flu mice. FOV size ~8,600 µm^2^ (**A**) and ~4,300 µm^2^ (**B**). *P < 0.05, **P < 0.01. Data in **C**, **D**, and **F** are shown as mean ± SEM and compared using Students’ *t* test. *n* = 5 mice per group and ~6–8 FOVs per mouse (**C**–**G** and **I**).

qFILM revealed in situ formation of intravascular NPAs that were more numerous, larger in size, and more enriched with platelets in the lung of CS+Flu compared with RA+Flu mice at day 4 after IAV infection. As shown by the representative FOV in Figure 3A and Supplemental Video 7, a neutrophil bound to platelets (marked with an arrowhead) can be seen crawling intravascularly, opposite to the direction of blood flow and toward a large NPA (marked with a dashed ellipse) composed of 4 neutrophils bound to platelets at 0 minutes in the lung microcirculation of a CS+Flu mouse. Over the next 3–6 minutes, the neutrophil moved closer to the large NPA, more platelets accumulated in the large NPA (green fluorescence increases), and more neutrophils (decorated with platelets) arrived in the same FOV. The neutrophil joined the NPA at 10 minutes, resulting in a larger NPA. Figure 3B and Supplemental Video 8 show a second representative example demonstrating in situ formation of a large NPA in the lung microcirculation of a CS+Flu mouse. A neutrophil bound to platelets (marked with an arrowhead in Figure 3B) is shown crawling intravascularly over 10 minutes to join an existing NPA (marked with dashed ellipse in Figure 3B), resulting in a larger NPA. The number of neutrophils crawling toward an NPA per FOV (size = 65,000 μm^2^) was significantly higher (Figure 3C) and the crawling velocity of these neutrophils was significantly lower (Figure 3, D and E) in the lung of CS+Flu compared with RA+Flu mice at day 4 after infection. qFILM data were also analyzed to estimate average numbers of NPAs per FOV and average size of NPAs (μm^2^) in the lung microcirculation that were 2-fold higher (significantly) in CS+Flu compared with RA+Flu mice (Figure 3, F and G). Remarkably, a majority of NPAs in the lung microcirculation of CS+Flu mice were composed of neutrophils attached to a large number of platelets (platelet-rich NPAs). Three representative examples of large platelet-rich NPAs (abundance of green fluorescence) in the lung of CS+Flu mice are shown in Figure 3H. In contrast, relatively smaller NPAs with few attached platelets were observed in the lung microcirculation of RA+Flu mice (Supplemental Figure 4). Indeed, the percent FOVs with at least 1 such platelet-rich NPA was significantly higher (2-fold) in the lung of CS+Flu compared with RA+Flu mice (Figure 3I).

### Platelet-rich NPAs promote severe pulmonary ischemia in CS+Flu mice.

**Figure 4:**
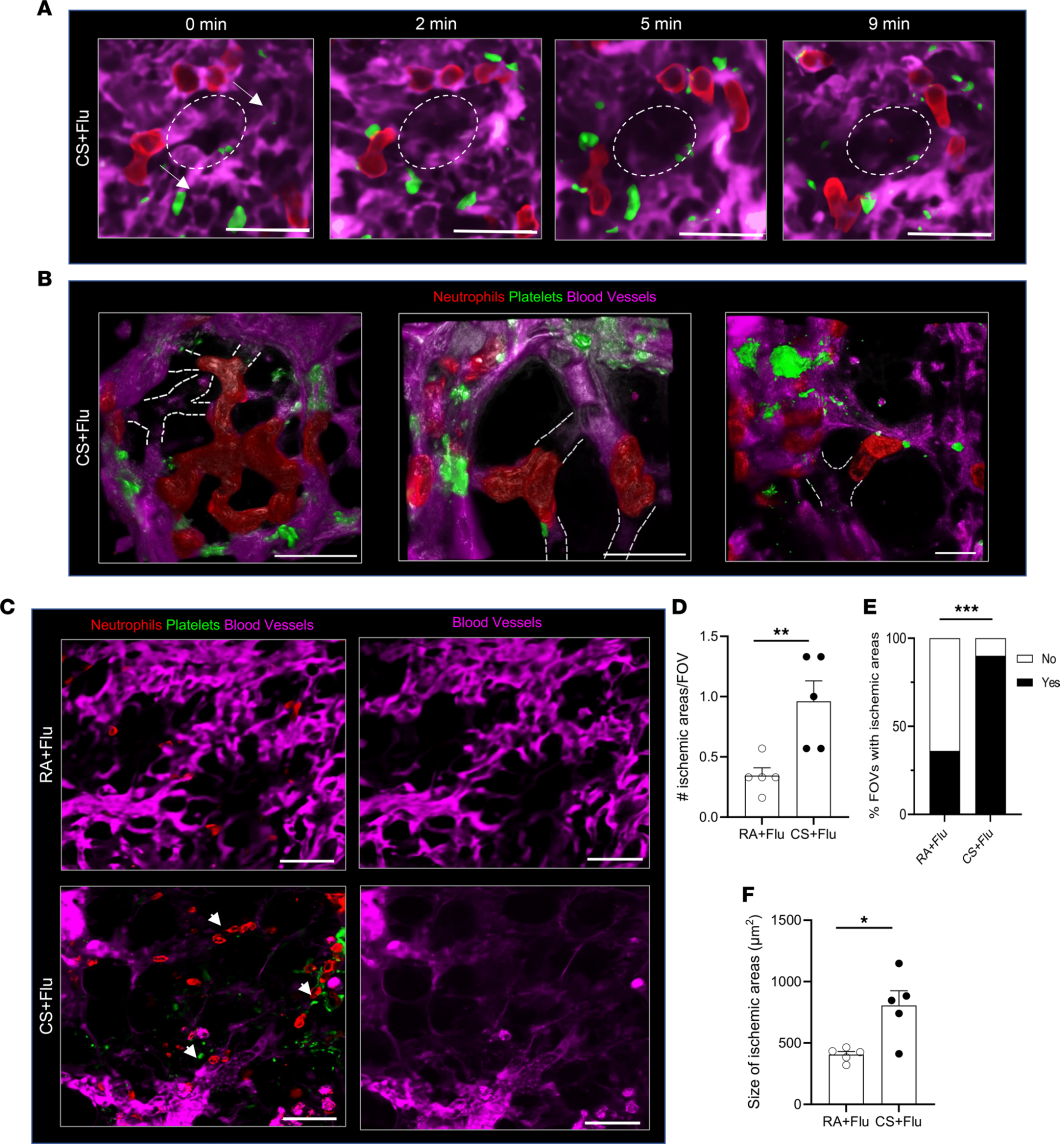
Platelet-rich NPAs promote severe pulmonary ischemia in CS+Flu mice. Mice were exposed to CS or RA for 4 weeks, followed by inoculation with flu, and qFILM was used to assess thrombo-inflammation in the lung at 4 days after infection. Microcirculation (pseudocolored purple), neutrophils (red), and platelets (pseudo-colored green). (**A**) Cropped qFILM images of the same FOV in the lung of a CS+Flu mouse shown at 4 different time points. Four neutrophils decorated with platelets crawl intravascularly (direction shown by arrows) over 9 minutes to form an NPA, which occludes a pulmonary microvessel (purple vascular dye disappears in the dashed ellipse). Time points relative to the first frame at 0 seconds. Refer to Supplemental Video 9. (**B**) Three representative qFILM images showing occlusion of lung microvessels (lack of blood flow evident by the absence of purple fluorescence in the microvessels marked with dotted lines). (**C**) Representative qFILM images of the lung of RA+Flu (top row) and CS+Flu (bottom row) mouse showing ischemic areas (dark regions without purple vascular dye). NPAs marked by arrowheads. Images in the right column show only the vascular dye (purple) channel of the respective 3-color images in the left column. Scale bars: 20 µm. qFILM data was analyzed to compare (**D**) number of ischemic areas per field of view (FOV), (**E**) percent FOVs with ischemic areas, and (**F**) size of ischemic areas in the lung of RA+Flu and CS+Flu mice. Data in **D** and **F** are shown as mean ± SEM and compared using Students’ *t* test. Data in **E** are shown as percentages and compared using χ^2^ distribution test. *n* = 5 mice per group and ~6–8 FOVs per mouse. *P < 0.05, **P < 0.01, ***P < 0.001. FOV size, 6,400 µm^2^.

Time series of cropped qFILM images (Figure 4A and Supplemental Video 9) reveal 4 neutrophils (red) decorated with platelets (green) crawling intravascularly (direction shown by arrows) in the lung microcirculation (purple) of a CS+Flu mouse (at day 4 after IAV infection) to form an NPA over a 9-minute period. At 9 minutes, the NPA occludes a pulmonary microvessel (marked with dashed ellipse) resulting in the loss of blood flow (purple vascular dye disappears in the dashed ellipse). Three-dimensional (3D) views of 3 representative examples of lung microvessel occlusion by NPAs in CS+Flu mice are shown in Supplemental Videos 10–12 and Figure 4B. This pathological process of microvascular occlusion led to more numerous and much larger ischemic areas within the lung of CS+Flu compared with RA+Flu mice (Figure 4C). Representative qFILM FOVs shown in Figure 4C reveal much larger ischemic areas (dark regions lacking purple vascular dye, suggestive of the absence of blood flow) associated with platelet-rich NPAs (arrowheads) in the lung of a CS+Flu (bottom row) compared with an RA+Flu (top row) mouse. The quantitative analysis of qFILM data over several mice revealed that the average number of ischemic areas per FOV (Figure 4D), percent FOVs with at least 1 ischemic area (Figure 4E), and the average size of ischemic areas (Figure 4F) were significantly (2-fold) higher in the CS+Flu compared with RA+Flu mice.

### Pulmonary ischemia leads to severe vascular leakage in the lung of CS+Flu mice.

**Figure 5:**
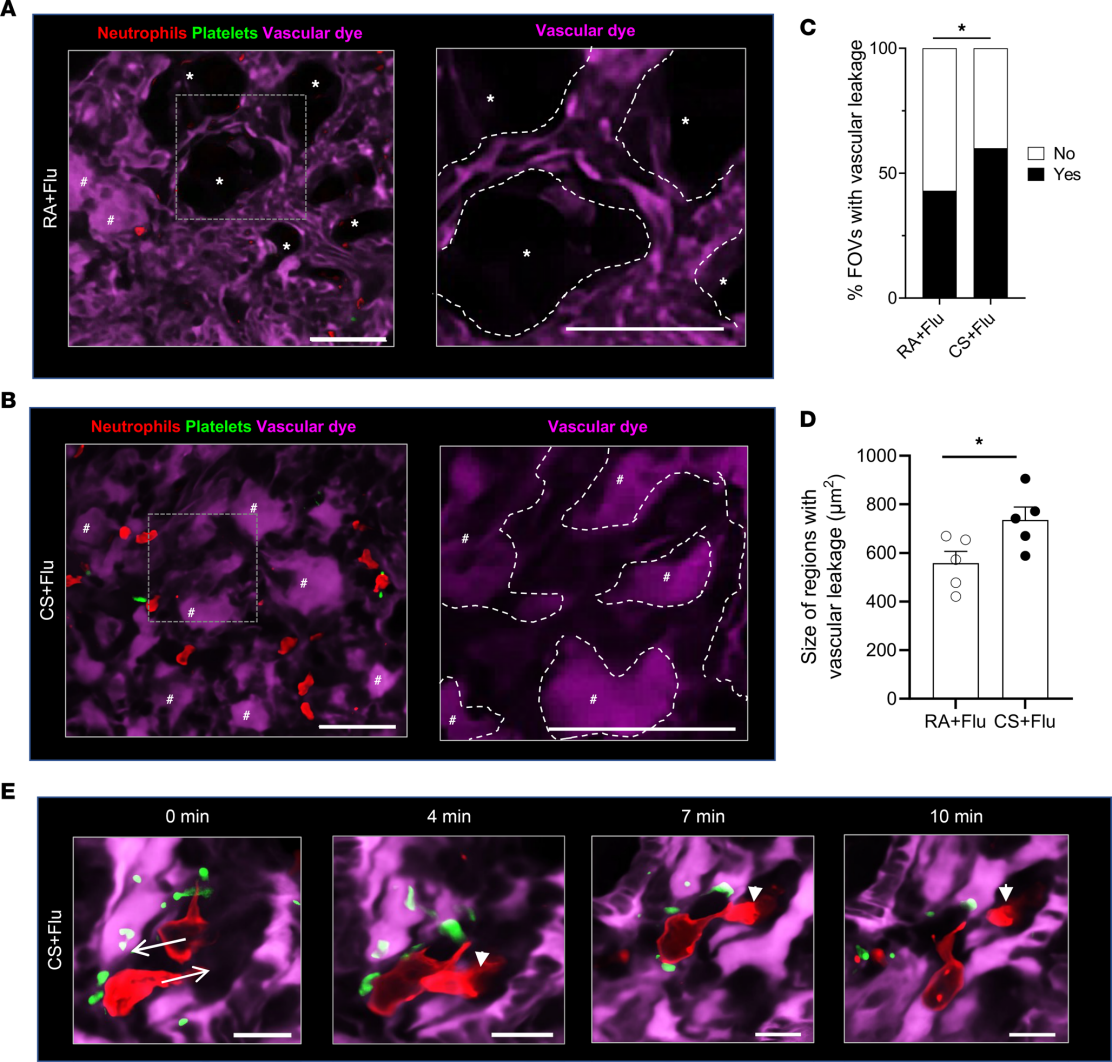
Pulmonary ischemia leads to severe vascular leakage in the lung of CS+Flu mice. Mice exposed to CS or RA for 4 weeks followed by inoculation with Flu, and qFILM used to assess thrombo-inflammation in the lung at 4 days after infection. Microcirculation (pseudocolored purple), neutrophils (red), and platelets (pseudocolored green). qFILM images of the lung microcirculation in an (**A**) RA+Flu and (**B**) CS+Flu mouse showing alveolar air spaces with (#) or without (*) vascular leakage (presence of purple vascular dye in air spaces). Right panels show magnified view of the dashed box in left panels. Dashed contours in right panels mark the walls of the pulmonary microvessels bordering the alveolar air spaces. Presence (**A**; right panel) and absence (**B**; right panel) of vascular dye between the dashed contours suggests the presence or absence of blood flow in microvessels. Refer to Supplemental Videos 13 and 14. Scale bars: 50 µm (left panels) and 20 µm (right panels). qFILM images were analyzed to compare (**C**) percent field of views (FOVs) with vascular leakage and (**D**) size of vascular leakage areas in the lung of RA+Flu and CS+Flu mice. Data in **C** are shown as percentages and compared using χ^2^ distribution test. Data in **D** are shown as mean ± SEM and compared using Students’ t test. *n* = 5 mice/group and ~6–8 FOVs per mouse. *P < 0.05. FOV size, ~65,000 µm^2^. (**E**) Cropped qFILM image of the same FOV in the lung of a CS+Flu mouse show 2 neutrophils crawling intravascularly in opposite directions (shown by arrows). One neutrophil crawls to the left; meanwhile, the second neutrophil crawls to the right and transmigrates into the air space. Arrowhead marks the disappearing tail of the emigrating neutrophil. Refer to Supplemental Video 15. Scale bar: 10 µm.

qFILM revealed more numerous and substantially larger areas with alveolar flooding in the lung of CS+Flu compared with RA+Flu mice (at day 4 after IAV infection) that were predominantly localized in the regions with pulmonary ischemia (Figure 5, A and B). Representative qFILM FOVs show leakage of vascular dye (purple) in only a few alveoli in an RA+Flu mouse lung (Figure 5A, left panel), while most alveoli appear to be flooded in the CS+Flu mouse lung (Figure 5B, left panel). In Figure 5, A and B, flooded alveoli are visible as purple islands, while alveoli lacking vascular leakage are visible as black polygons. The right panels (Figure 5, A and B) show a high-magnification view of the regions marked by a dotted box in the respective left panels of Figure 5, A and B. As shown in these high magnification images, unflooded alveoli were surrounded by perfused microvessels in the RA+Flu mouse lung (erythrocytes visible as rapidly transiting dark cells in Figure 5A [right panel] and Supplemental Video 13), while, flooded alveoli were surrounded by ischemic microvessels (vascular dye absent) in the CS+Flu mouse lung (Figure 5B [right panel] and Supplemental Video 14), suggesting that the alveolar flooding in CS+Flu mice is associated with the development of severe pulmonary ischemia. Quantitative analysis of qFILM data reveal that percent FOVs with vascular leakage (Figure 5C) and the mean vascular leakage area per FOV (Figure 5D) were significantly higher in the lung of CS+Flu compared with RA+Flu mice. Remarkably, alveolar flooding in the CS+Flu mice was also accompanied by the recruitment of numerous NPAs into the alveolar air space, and the number of recruited NPAs was higher in CS+Flu compared with RA+Flu mice (data not shown). The time series of qFILM images (Figure 5E and Supplemental Video 15) demonstrate 2 neutrophils (red) decorated with platelets (green) crawling in opposite directions (marked by arrows at 0 minutes) within the pulmonary microcirculation (purple) of a CS+Flu mouse. One neutrophil can be seen crawling intravascularly to the left over a 10-minute period. Meanwhile, the second neutrophil crawls to the right and then gradually emigrates into the alveolar air space (arrowhead marks the disappearing tail of the emigrating neutrophil at different time points). As shown in Supplemental Figure 5, the platelet-rich NPAs, ischemia, and vascular leakage were significantly resolved by day 14 after infection in CS+Flu mice.

## Discussion

Although cigarette smoking is a major risk for increased morbidity and mortality among patients with the flu (9–12), how prior exposure to CS promotes flu severity remains poorly understood. To address this, we used a 2-hit mouse model involving CS exposure followed by a mild nonlethal dose of intranasal IAV, which led to severe lung injury accompanied by excessive weight loss and acute hypoxemia in mice preexposed to CS, but it led to mild lung injury in mice preexposed to RA. Real-time intravital microscopy of the lung revealed that the development of acute severe respiratory dysfunction in CS+Flu mice was associated with the accumulation of numerous large NPAs in the pulmonary microcirculation within 2 days following IAV infection; however, such large NPAs were absent in RA+Flu mice even at day 4 after IAV infection.

Remarkably, the NPAs in the lung of CS+Flu mice were not only more numerous and larger in size but were also more enriched with platelets compared with those in the lung of RA+Flu mice. These platelet-rich NPAs formed in situ within the pulmonary microcirculation and grew larger over time, leading to occlusion of pulmonary microvessels and loss of blood flow in large areas of the lung in CS+Flu mice. Pulmonary ischemia is known to cause the ischemia-reperfusion injury, neutrophil respiratory burst, and endothelial reactive oxygen species generation that may contribute to blood-air barrier disruption (34, 35). Indeed, pulmonary ischemia was followed by the infiltration of NPAs into the air spaces and alveolar flooding in large areas of the lung, leading to the development of severe hypoxemia in CS+Flu mice. Taken together, these findings suggest, for the first time to our knowledge, that the early onset of pulmonary microvasculature occlusion by platelet-rich NPAs contributes to CS induced flu severity in mice.

Our current findings lead to several questions that may inspire future studies. First, emerging evidence suggests that upregulation of innate-immune pathways in neutrophils and platelets correlates with flu severity (6, 15, 18, 36, 37), but the pathways promoting platelet-rich NPA formation in the setting of CS-induced flu severity remain to be identified. The molecular mechanism underlying CS+Flu–induced neutrophil and/or platelet activation needs to be investigated in future studies. Second, although our current study did not investigate the role of coagulation in promoting CS induced flu severity, cigarette smoking has been associated with an increased risk of coagulopathy (38, 39). Third, our 2-hit model using a short-term CS exposure may not translate how an underlying chronic condition such as chronic obstructive pulmonary disease (COPD) would affect flu severity; therefore, more elaborate mouse models would be required to address this in future studies. Fourth, it remains to be identified how CS-induced dysfunction in airway epithelial and pulmonary vascular endothelial cells may contribute to CS+Flu–induced severe pulmonary thrombo-inflammation (13, 40–42).

Notwithstanding these limitations, our current study uses real-time in vivo lung imaging in live mice for the first time to our knowledge to reveal the kinetics of the acute severe thrombo-inflammatory response in the pulmonary microcirculation, which contributes to the development of severe lung injury in mice exposed to CS prior to a mild IAV infection. These findings suggest that identification of innate immune pathways promoting the CS- and Flu-induced severe thrombo-inflammatory response could lead to the development of new therapies for attenuating flu severity in smokers.

## Methods

Supplemental Methods are available online with this article.

### Reagents.

Alexa Fluor 546 (AF546) rat anti–mouse Ly6G mAb (clone 1A8) and Violet 450 (V450) rat anti–mouse CD49b mAb (clone DX5) were purchased from BioLegend. FITC dextran (MW 70 KDa) was purchased from Thermo Fisher Scientific. RLT+ lysis buffer (catalog 1030963) and RNeasy Plus Micro Kit (catalog 74034) were purchased from Qiagen. High-capacity cDNA reverse transcription kit (catalog 4368814) and PowerUp SYBR Green Master Mix (catalog A25742) were purchased from Thermo Fisher Scientific. Ambion nuclease-free water (catalog AM9906) was purchased from Invitrogen. Quantitative PCR (qPCR) primers were purchased from Integrated DNA Technologies. Sterile saline solution (catalog 65207-807-60) was purchased from Nova-Tech. Gibco PBS without Ca^2+^ and Mg^2+^ (catalog 10010072), buffered 10% formalin (catalog SF100-4), and absolute ethanol (catalog BP2818100) were purchased from Thermo Fisher Scientific. AnaSed Xylazine (catalog 59399-111-50) was purchased from Akorn Pharmaceuticals. Ketamine was purchased from Covetrus.

### Mice.

WT male mice (approximately 8 weeks) on a C57BL/6J background purchased from The Jackson Laboratory were used in this study. Mice were randomly divided into 3 groups: RA+Veh (exposed to RA followed by inoculation with sterile PBS), RA+Flu (exposed to RA followed by inoculation with the flu virus as described below), and CS+Flu (exposed to CS for 4 weeks as described below followed by 3-day gap and then inoculation with the flu virus). Mice were maintained under pathogen-free conditions at the University of Pittsburgh, Division of Laboratory Animal Resources (DLAR) facility in Animal Biosafety Level 2 (ABSL2) zone with unlimited access to autoclaved water and food.

### CS exposure.

Mice were whole-body exposed to CS for 4 weeks using an automated closed-chamber inExpose compact-inhalation CS exposure unit purchased from Scireq, which was controlled by inExpose 6.1 software (Scireq) installed on a PC. Research cigarettes (1R6F) purchased from the Center for Tobacco Reference (University of Kentucky, Lexington, Kentucky, USA) were used in the inExpose unit. Mice were placed in the CS exposure chamber of the inExpose unit, and the inExpose 6.1 software was used to enable 1 puff of cigarette (2 seconds long with a volume of 35 mL) per minute, which resulted in 2.5% (vol/vol of smoke/air) leading to 350 μg total particulate matter (TPM)/L (equivalent to 18.4 μg nicotine/L) in the chamber throughout the duration of the exposure as described in prior studies (43, 44). Mice were placed in the CS-exposure chamber 5 days per week for 30 minute per day in the first week (to allow acclimatization) followed by 1 hour per day in the remaining 3 weeks. After 4 weeks, mice were kept in standard RA conditions for 3 days, followed by intranasal IAV or sterile PBS (vehicle) inoculation.

### IAV instillation.

Mouse-adapted influenza A/PR/8/34 H1N1 virus (IAV) was propagated in chicken eggs as described elsewhere (45). Mice were intranasally administered about 5 × 10^4^ PFU of IAV (suspended in 60 μL of sterile PBS) or 60 μL of sterile PBS (vehicle) under isoflurane anesthesia. Infected mice were housed in ABSL2 facility up to 14 days. qFILM studies were conducted in mice 1–14 days after IAV infection. ALI was assessed by harvesting lungs for histology at day 9 after IAV infection. Body weight and blood oxygen saturation levels were measured daily after IAV infection in mice. Lung viral burden was assessed in the middle lobe of the right lung by qPCR.

### Assessment of lung injury.

Lungs were harvested from mice, fixed in 10% formaldehyde for 24 hours, and then stored in 70% ethanol at room temperature as described elsewhere (46). Tissue sections were stained with H&E, and images were collected using an automatic high-resolution microscopic scanner by Histowiz Inc. Histological analysis was performed by a modified extensive scoring system (47), based on the guidelines of American Thoracic Society for assessment of ALI in mice (32, 33). The following 5 criteria were used to score lung injury: hemorrhage, edema, vascular congestion, alveolar wall thickening, and percent injured area. Sections were scored by 2 independent investigators as follows: 0, absent; 1, mild; 2, moderate; 3, severe; 4, very severe. Individual scores for a single mouse were calculated from the mean score from 5 random focal areas (magnification, ×20) with the score ranging from 0 to 4 and averaged from 2 investigators blinded to scoring. The mean injury score was determined for lungs from at least 10 animals per experimental group to generate a cumulative lung injury score. Additionally, the MouseOx pulse-oximeter (Starr Life Sciences) was used to measure real-time percent blood oxygen saturation in a mouse as described previously (48).

### Assessment of lung viral burden.

Mice were sacrificed, and the middle lobe of the right lung was snap frozen in the liquid nitrogen. Lung tissue was homogenized using gentle MACS dissociation unit (Miltenyi Biotec) and lysed in RLT lysis buffer enriched with 1% of β-mercaptoethanol. Total RNA was isolated from the lung tissue using the RNeasy Plus Micro Kit (Qiagen) according to vendors instructions. In total, 1 μg of total extracted RNA was used as a template to make first-strand complementary DNA (cDNA) using high-capacity cDNA reverse transcription kit (Applied Biosystems). qPCR analyses were performed using Applied Biosystems StepOnePlus Real-Time PCR System with PowerUp SYBR Green Master Mix in a 40-cycle–long PCR to assess levels of IAV M1 protein. The relative amount of the M1 protein gene expression was normalized relative to the level of *RNA18s* (housekeeping) gene and shown as relative expression in arbitrary units. The primers for M1 viral protein were as follows: forward 5′-GGACTGCAGCGTAGACGCTT-3′, reverse: 5′-CATCCTGTTGTATATGAGGCCCAT-3′. The primers for *RNA18s* were as follows: forward 5′-GGACCAGAGCGAAAGCATTTGCC-3′, reverse: 5′-TCAATCTCGGGTGGCTGAACGC-3′.

### Plaque-forming assay.

MDCK cells (ATCC CCL-34) were initially seeded at a density of 1 × 10^6^ cells per well in flat-bottom, 6-well tissue culture plates. The next day, the MDCK growth medium was removed, and the cells were washed twice with plaque assay wash medium (DMEM). Subsequently, 200 μL of DMEM containing 10-fold serial dilutions of the supernatant from a homogenized lung sample were added to the wells. After 1-hour incubation, 2 mL of 0.8% agarose overlay containing plaque assay medium was added to each well. The plates were then incubated for 72 hours and fixed with a solution of 4% paraformaldehyde (PFA) in PBS for 1 hour. Following fixation, the plates were stained with 0.5% (w/v) Crystal Violet and washed twice with water. The plaques were counted after plates were dried overnight at room temperature. The final calculation is based on the formula: pfu/g = no. of plaques × (dilution factor/weight of lung [g]).

### qFILM.

qFILM has been used widely for in vivo assessment of thrombo-inflammation in the pulmonary microcirculation (25, 27, 28). In the current study, qFILM was used to detect in situ formation of NPAs in the lung microcirculation, pulmonary ischemia, and vascular leakage in the intact lung of live mice. The qFILM experimental setup and approach has been described elsewhere in detail (25, 26, 28, 29). Briefly, qFILM was performed with a Nikon multi-photon-excitation (MPE) fluorescence microscope (Nikon Instruments Inc.) using an excitation wavelength of 850 nm and an apochromatic long working distance (APO LWD) 25× water immersion objective with 1.1 NA. Time-series of 2D qFILM images were collected at ~15 frames per second (fps) using a resonant scanner or ~2 fps using Galvano-scanner. Each FOV was 256 μm × 256 μm (~65,536 μm2) with a resolution of ~0.5 μm per pixel in the *x*–*y* plane. Fluorescent light received from the sample was collected by different detectors using series of band pass filters: 450/20 nm (detector 1 for collection of V450 or pacific blue), 525/50 nm (detector 2 for collection of FITC), and 576/26 nm (detector 3 for collection of AF546). Prior to thoracic surgery, mice were anesthetized with an i.p. injection of a cocktail containing 100 mg/kg ketamine HCl and 20 mg/kg xylazine. Tracheotomy was performed to facilitate mechanical ventilation with ~95% O_2_ and supply maintenance anesthesia (1.5% isoflurane). The left lung was surgically exposed by serrating 3 ribs and immobilized against a coverslip using a vacuum-enabled micromachined device as described elsewhere (26, 29). Just prior to imaging, ~125 μg/mouse FITC-dextran, 12 μg/mouse AF546-conjugated anti-Ly6G mAb, and 7 μg/mouse V450-conjugated anti–mouse CD49b mAb were injected via the femoral vein for visualization of the pulmonary microcirculation, neutrophils, and platelets, respectively. qFILM was performed on a mouse for a total period of 60 minutes, and the presence or absence of NPAs, size of NPAs, presence or absence of vascular leakage, NPA infiltration into the air space, and number/size of areas with absence of blood flow in the lung were assessed for ~5–10 minutes in each FOV (total of 6 FOVs per mouse). Time series of qFILM 2D images were processed and analyzed using Nikon’s NIS-Elements software as described previously (25, 26, 29). First, image subtraction algorithm was performed to remove autofluorescence and bleed through between channels. Second, signal/noise ratio was improved by using a median filter algorithm followed by smoothing and denoising algorithms. Third, each channel was pseudocolored as follows: microcirculation as purple, neutrophils as red, and platelets as green to enhance contrast and facilitate visualization. NPAs were defined as neutrophils bound to platelets. Platelet-rich NPAs were defined as few neutrophils (1 or 2 neutrophils) decorated with platelets aggregate. Vascular leakage was defined by the presence of vascular dye (pseudocolored purple) within the alveolar air spaces of the lung. Ischemic areas were defined as regions (>50 μm^2^) in the lung with no apparent blood flow, evident by the lack of vascular dye (purple) in the pulmonary microvessels.

### Statistics.

Means were compared using unpaired 2-tailed Student’s *t* test or 1-way ANOVA followed by Tukey post hoc test for multiple comparisons. Percentages were compared using χ^2^ distribution test. Comparative statistical analysis was performed by 1-way ANOVA with Bonferroni correction. Data were analyzed using GraphPad Prism 10.0 software.

### Study approval.

All animal experiments were approved by the IACUC at the University of Pittsburgh, VERSITI Blood Research Institute, and the Medical College of Wisconsin (Milwaukee, Wisconsin, USA).

### Data availability.

Data associated with the manuscript and supplemental materials are available as Supporting Data Values file.

## Author contributions

TWK designed the experiment; performed CS exposure, organ collection, and qFILM experiments; analyzed the data; and wrote the manuscript. TB and RV were involved in qFILM data acquisition and analysis. XL performed organ collection. OK maintained mice colonies. RKD was involved in qPCR experiments. KB and LZ performed plaque-forming assays. KMR provided the virus and participated in experimental design. BJM, TPS, and SCW participated in experimental design. TN and PS were responsible for experimental design, manuscript writing, and project supervision. TWK, TN, and PS wrote the manuscript, with consultation and contribution from all coauthors.

## Supplementary Material

Supplemental data

Supplemental video 1

Supplemental video 10

Supplemental video 11

Supplemental video 12

Supplemental video 13

Supplemental video 14

Supplemental video 15

Supplemental video 2

Supplemental video 3

Supplemental video 4

Supplemental video 5

Supplemental video 6

Supplemental video 7

Supplemental video 8

Supplemental video 9

Supporting data values

## Version 1 12/07/2023

In-Press Preview

## Version 2 01/23/2024

Electronic publication
