# Supplementary material for: Lung microvascular occlusion by platelet-rich neutrophil-platelet aggregates promotes cigarette smoke–induced severe flu
Source: JCI Insight. 2024 Jan 23;9(2):e167299. doi: 10.1172/jci.insight.167299 (PMC10906226; doi:10.1172/jci.insight.167299)
Supplement: Supplemental data [file jciinsight-9-167299-s125.pdf]

SUPPLEMENTAL FIGURES

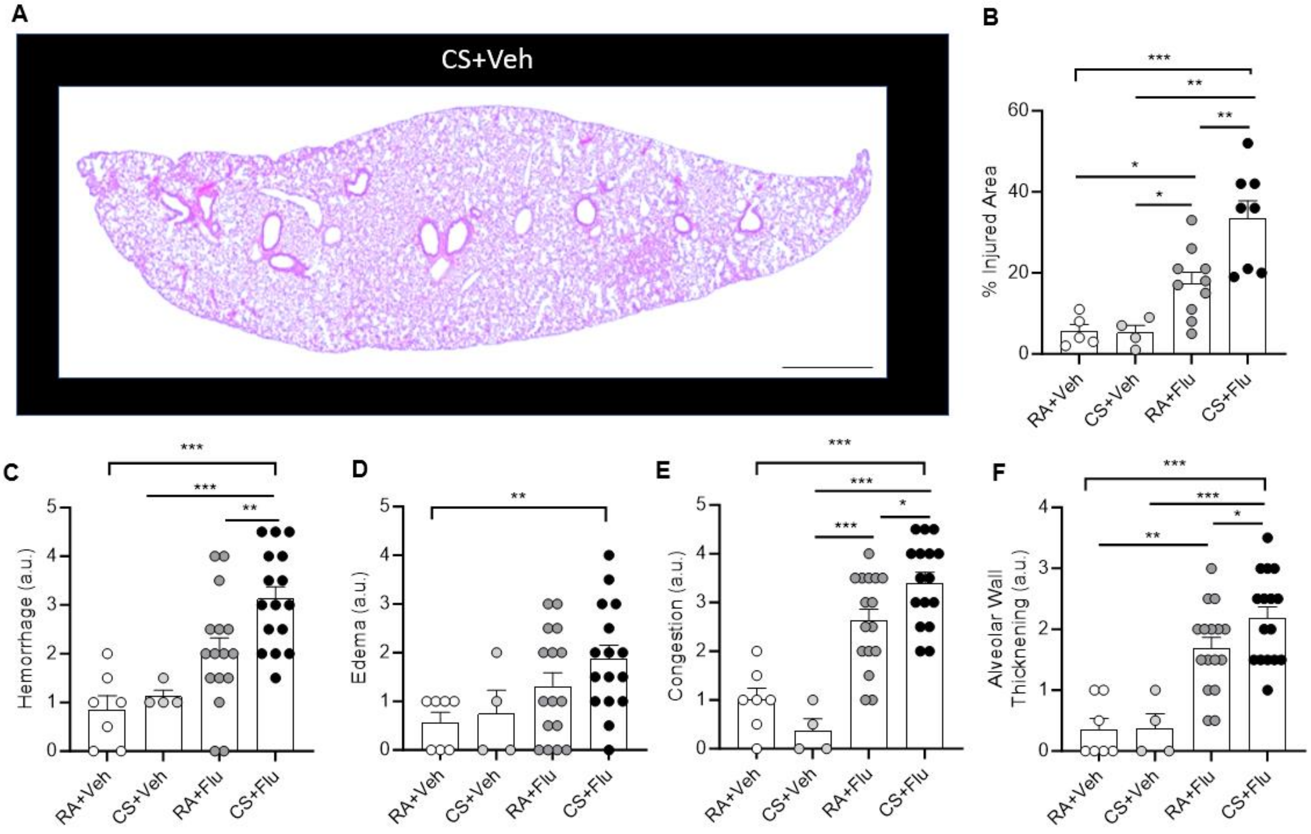

Supplemental Figure 1. Lung injury is absent in mice exposed to four weeks of CS only (without IAV infection). Wild type mice were exposed to CS for four weeks followed by intranasal administration of sterile PBS (vehicle). **(A)** Representative hematoxylin and eosin (H&E) stained histological section of the whole left lung of a CS+Vehicle mouse at day 9 post Vehicle treatment. Scale bar is 100µm. Lung histological sections were scored (refer to Methods for details) for **(B)** percentage of injured area and severity of **(C)** hemorrhage, **(D)** pulmonary edema, **(E)** vascular congestion, **(F)** alveolar wall thickening. N=3 to 16 mice per group. Data shown as Mean ± SE and compared using Students' t test. *p<0.05; **p<0.01; ***p<0.001.

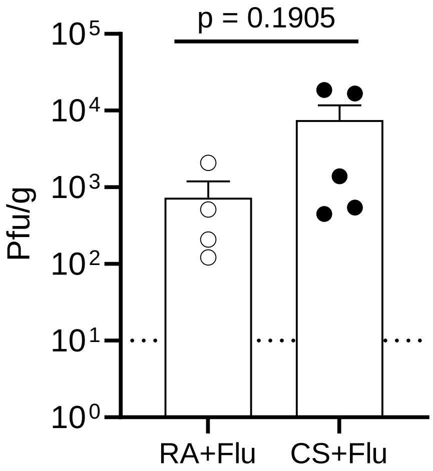

Supplemental Figure 2. Viral burden at day 9 post flu infection is not different in mice pre-exposed to cigarette smoke than room air. Mice were exposed to cigarette smoke (CS) or room air (RA) for four weeks followed by intranasal administration of a mild dose of mouse adapted influenza A virus (A/PR/8/34 H1N1). Experimental scheme shown in Figure 1A. At day 9th post IAV infection, viral burden was assessed in whole lungs using plaque assay and plotted as plaque forming units per gram (pfu/gm) of the lung tissue. The viral burden was not significantly different ($p=0.1905$; N=4-5 mice per group). Data shown as Mean ± SE. Data compared using Students' t test.

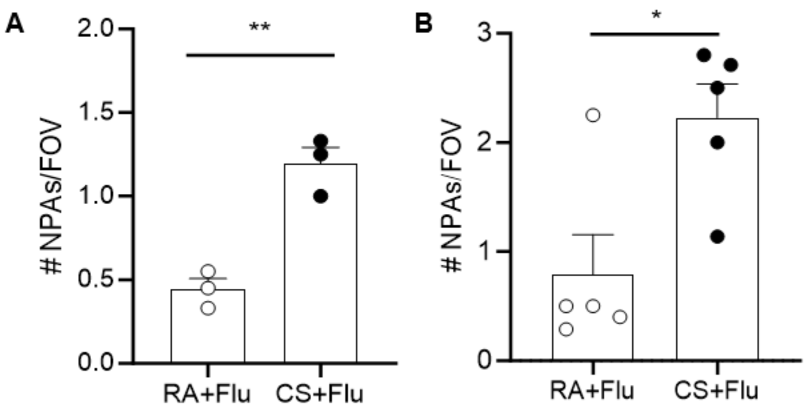

Supplemental Figure 3. Neutrophil-platelet aggregates were more abundant in the lung microcirculation of CS+Flu than RA+Flu mice. Experimental scheme shown in Figure 2A. Quantitative fluorescence intravital lung microscopy (qFILM) images were analyzed to compare number of neutrophil-platelet aggregates per field of view (#NPAs/FOV) in the lung microcirculation of CS+Flu and RA+Flu mice. #NPAs/FOV were significantly higher in the lung of CS+Flu than RA+Flu mice both at **(A)** day 2 and **(B)** day 4 post flu infection. Data shown as Mean ± SE. N=3-5 mice per group and 6 FOVs per mouse. * $p<0.05$; ** $p<0.01$. FOV size ~ 65000 μm^2. Data compared using Students' t test.

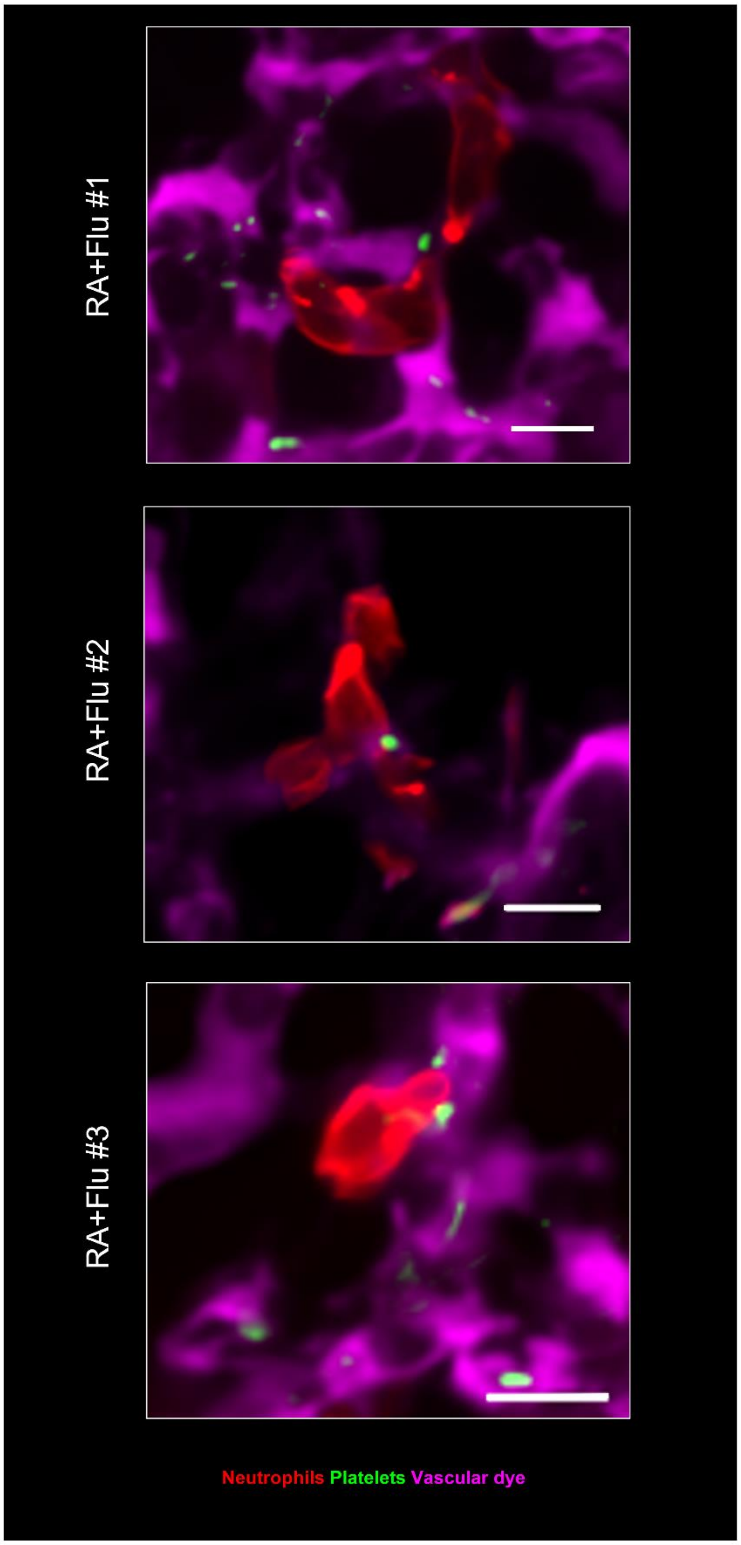

Supplemental Figure 4. Small neutrophil-platelet aggregates are present in the lung of RA+Flu mice. Experimental scheme shown in Figure 2A. Mice were exposed to room

air (RA) for 4 weeks followed by intranasal administration of a mild dose of mouse adapted influenza A virus (A/PR/8/34 H1N1) and quantitative fluorescence intravital lung microscopy (qFILM) was used to assess the lung microcirculation in live mice at 4 days post IAV infection. The microcirculation (pseudo-colored purple), neutrophils (red) and platelets (pseudo-colored green) were visualized *in vivo* by IV administration of FITC dextran, AF546-anti-mouse Ly6G Ab and Pacific blue-anti-mouse CD49b Ab, respectively. Three representative qFILM images showing small neutrophil-platelet aggregates (NPAs) composed of 1 or 2 neutrophils with few attached platelets in the lung microcirculation of RA+Flu mice. Scale bars are 10 µm.

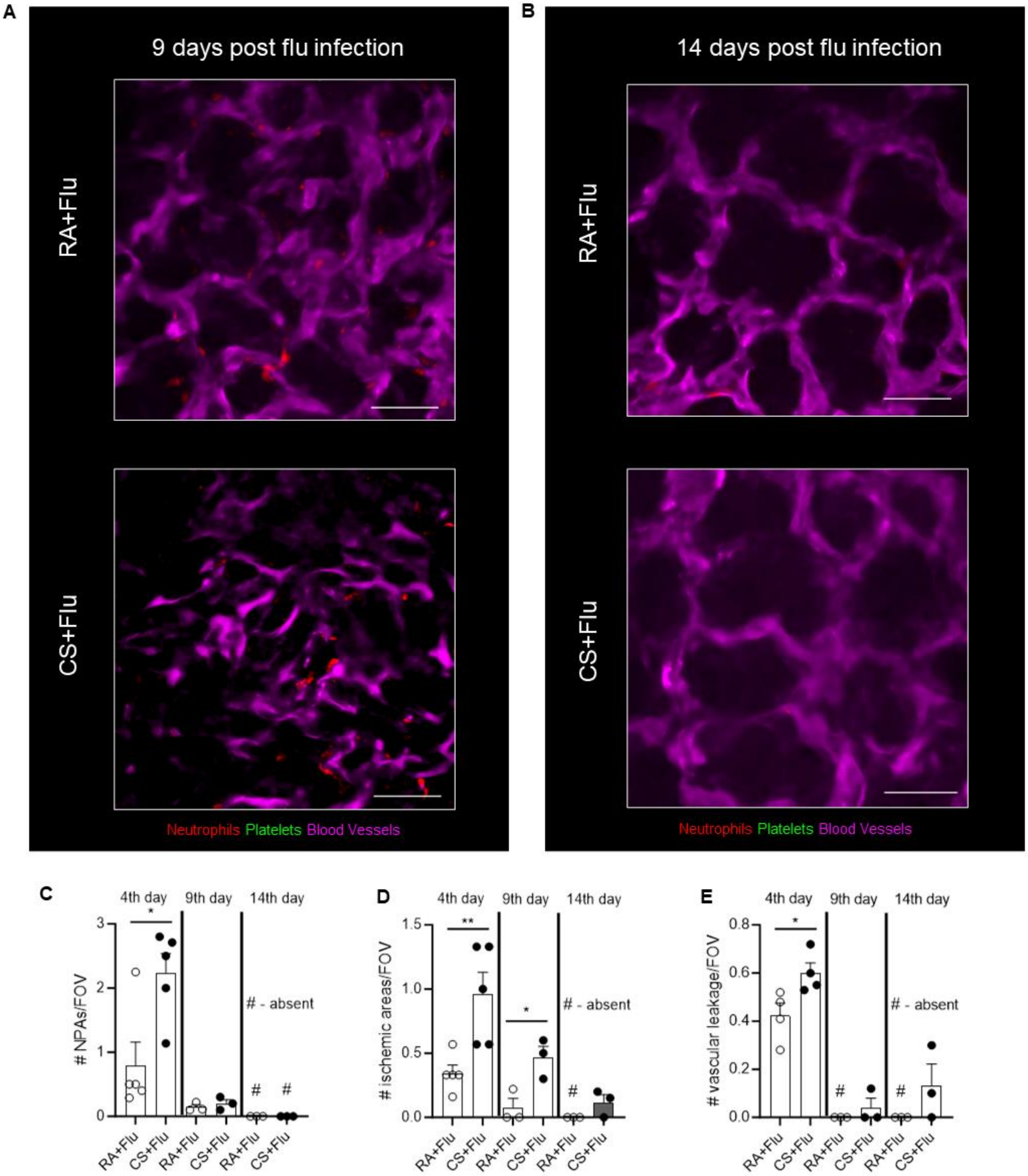
A
9 days post flu infection
RA+Flu
CS+Flu
Neutrophils Platelets Blood Vessels
B
14 days post flu infection
RA+Flu
CS+Flu
Neutrophils Platelets Blood Vessels
C
4th day
9th day
14th day
NPAs/FOV
# - absent
RA+Flu
CS+Flu
D
ischemic areas/FOV
# - absent
E
vascular leakage/FOV
# - absent

Supplemental Figure 5. NPAs, ischemia and vascular leakage are resolved in the lung of CS+Flu mice by day 14 post flu infection. Experimental scheme shown in Figure 2A. Mice were exposed to room air (RA) or cigarette smoke (CS) for 4 weeks followed by intranasal administration of a mild dose of mouse adapted influenza A virus (A/PR/8/34 H1N1) and quantitative fluorescence intravital lung microscopy (qFILM) was used to assess the lung microcirculation in live mice at 9 or 14 days post IAV infection. The microcirculation (pseudo-colored purple), neutrophils (red) and platelets (pseudo-colored green) were visualized *in vivo* by IV administration of FITC dextran, AF546-anti-mouse Ly6G Ab and pacific blue-anti-mouse CD49b Ab, respectively. Refer to Methods for details. Representative qFILM images of lung microcirculation in RA+Flu and CS+Flu mice are shown at **(A)** day 9 and **(B)** day 14 post IAV infection. Scale bars 50 µm. QFILM images were analyzed as described in Methods to compare **(C)** number of NPAs per FOV (#NPAs/FOV), **(D)** number of ischemic areas per FOV (#ischemic areas/FOV) and **(E)** number of vascular leakage areas per FOV (#vascular leakage/FOV) in the lung of RA+Flu and CS+Flu mice at day 4, 9 and 14 days post flu infection. Data shown as Mean ± SE and compared using Students' t test. N = 3-5 mice per group and 6 FOVs per mouse. * $p<0.05$, ** $p<0.01$. # denotes the absence of the events within the group. FOV size ~ 65000 μm^2.

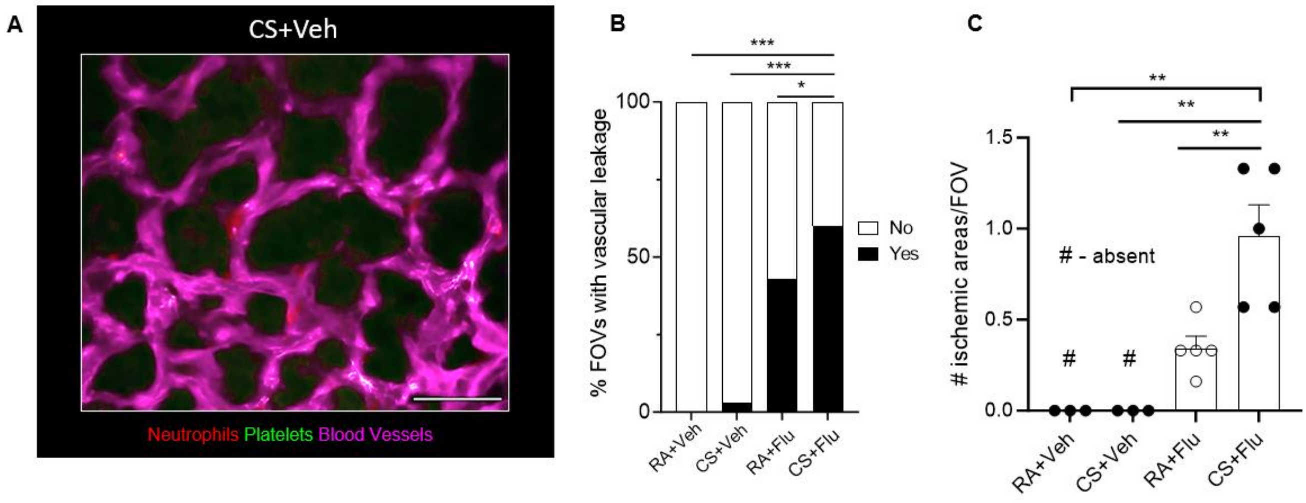

Supplemental Figure 6. Neutrophil-platelet aggregates, vascular leakage and ischemia are absent in the lung of mice exposed to CS only. Wild-type mice were exposed to CS for four weeks followed by intranasal administration of sterile PBS (vehicle) and quantitative fluorescence intravital lung microscopy (qFILM) was used to assess thrombo-inflammation in the lung of live mice at 4 days post vehicle (Veh) treatment. The microcirculation (pseudo-colored purple), neutrophils (red) and platelets (pseudo-colored green) were visualized *in vivo* by IV administration of FITC dextran, AF546-anti-mouse Ly6G Ab and V450-anti-mouse CD49b Ab, respectively. Refer to Methods for details. **(A)** A representative qFILM image shows absence of neutrophil-platelet aggregates (NPAs) in the lung of a mouse exposed to CS+Veh. The quantitative analysis of qFILM data (refer to Methods for details) revealed absence of **(B)** vascular leakage and **(C)** ischemic areas in the lung of CS+Veh mice. Data in D shown as percentages and compared using χ^2 distribution test. Data in C shown as Mean ± SE and compared using Students' t test. N=3-5 mice per group and ~6-8 FOVs per mouse. * $p<0.05$. ** $p<0.01$. *** $p<0.001$. FOV size ~ 65000 μm^2.

SUPPLEMENTAL METHODS

Quantification of the crawling velocity of neutrophils

The crawling velocity of neutrophils was estimated by analyzing time series of 2D qFILM images using Nikon NIS-Elements software. For precise estimation of the velocity (µm/min), semi-automatic polyline tracking function in NIS-Elements was used and the total length of crawled path was measured over a known time period. Only neutrophils present within a FOV for at least 5 minutes (>300 sec) were included in this analysis.

Three dimensional imaging of NPAs within the lung of CS+Flu mice

Surgical preparation of mice and general parameters of Nikon multi-photon-excitation (MPE) fluorescence microscope was described in the Materials and Methods section. For three dimensional (3D) imaging, mice were injected via femoral vein with ~125 µg/mouse FITC-dextran, 12 µg/mouse AF546-conjugated anti-Ly6G mAb and 7 µg/mouse V450-conjugated anti-mouse CD49b mAb for visualization of the pulmonary microcirculation. The presence of NPAs in the microcirculation was confirmed using x-y scanning of the lung microcirculation and at least 3 NPAs/mouse were selected for 3D imaging. Each 3D imaged area was 8µm x 40µm x 20µm (total volume of ~6400µm^3) and number of pictures per plane was set to 3, then merged into single plane view. To capture longitudinal NPAs, 3D image Z-stack loop was set to 200 planes with Z-step size of 0.1µm and fixed calibration of 0.13 µm/px. Z-plane scanning procedure was performed bidirectionally with scanning speed of 10 frames per seconds with 4x scanner zoom. QFILM 3D-images were processed and analyzed using Nikon's NIS-Elements software as described in the Materials and Methods section.
